# Anti‐cancer effect of entacaponeon esophageal cancer cells via apoptosis induction and cell cycle modulation

**DOI:** 10.1002/cnr2.1759

**Published:** 2022-12-19

**Authors:** Fahimeh Ramedani, Seyyed Mehdi Jafari, Marie Saghaeian Jazi, Zeinab Mohammadi, Jahanbakhsh Asadi

**Affiliations:** ^1^ Metabolic Disorders Research Center Golestan University of Medical Sciences Gorgan Iran; ^2^ Stem Cell Research Center Golestan University of Medical Sciences Gorgan Iran

**Keywords:** entacapone, epitranscriptome, esophageal squamous cancer cells, FTO

## Abstract

**Background:**

Esophageal cancer (EC) is the sixth leading cause of cancer‐related death, despite many advances in treatment, the survival of patients still remains poor. In recent years, the N6‐methyladenosine (m6A) has been introduced as one of the most important modifications at the epitranscriptome level, with an important role in the mRNA regulation in various diseases, such as cancers. The m6A is regulated by different factors, including FTO as a demethylase. The m6A modification, especially through FTO overexpression has an oncogenic role in different cancer types such as EC. Recent studies showed that entacapone, a catechol‐o‐methyl transferase (COMT) inhibitor currently applied for Parkinson's disease, can inhibit FTO enzyme.

**Aims:**

In this study, we aimed to investigate the effect of entacapone as an FTO inhibitor on the m6A level and also apoptosis and cell cycle response in KYSE‐30 and YM‐1 of esophageal squamous cancer cell (ESCC) lines.

**Methods:**

Cell toxicity and IC50 of entacapone were evaluated using The MTT assay in YM‐1 and KYSE‐30 cells. Cells were treated into two groups: DMSO (control) and entacapone (mean IC_50_). Total RNA was extracted, and m6A levels were measured via the ELISA method. Subsequently, the apoptosis and cell cycle dys‐regulation were detected by annexin‐V‐FITC/PI staining and PI staining via flow cytometry.

**Results:**

Entacapone has the cytotoxicity effect on both esophageal cancer cell lines compared to normal PBMC cells. As well, entacapone treatment (140 μM) can induce apoptosis (KYSE‐30: 50%. YM‐1:22.6%) and has a modulatory effect on cell cycle progression in both YM‐1 and KYSE‐30 cells (*p*‐value<.05). However, no significant difference in the m6A concentration was observed.

**Conclusion:**

Our findings suggested that entacapone has the inhibitory effect on ESCC cell lines through induction of the apoptosis and modulation of the cell cycle without toxicity on the normal PBMC.

## 1 INTRODUCTION

Esophageal cancer (EC) is the eighth most common malignancy and sixth leading cause of cancer‐related death worldwide.^1^ Esophageal cancer is divided into two common histologic subtypes: esophageal adenocarcinoma (EAC) and esophageal squamous cell carcinoma (ESCC).^2^ In Asia, ESCC is the most frequent histologic subtype of esophageal cancer.^3^, ^4^, ^5^ In recent years, despite of the great improvements in treatment of ESCC patients, the prognosis of patients remains poor and overall survival rate is still low.^2^, ^6^

Cancer is considered as a complex disease and different environmental, genetic and epigenetic factors can play role in cancer development or progression. More recently, epitranscriptome changes particularly with N6‐methyladenine (m6A) modification, has been reported with association to multiple diseases, such as cancer.^7^, ^8^, ^9^ The m6A is the common chemical modification of messenger RNA (mRNA), involved in mRNA splicing, stability, transcription, splicing, localization, translation regulation process.^10^ The level of m6A modification in mRNAs is regulated by multiple proteins called, “writer,” “eraser”, and “reader” proteins.^11^, ^12^ The writers are consisted of methyl‐transferase complex, METTL3, METTL14 and WTAP.^13^ In contrast the eraser proteins, namely FTO and ALKBH5 have the role of m6A demethylation.^13^ The Reader protein family includes the YTH domain‐containing family proteins YTHDF1/2/3, YTHDC1, and YTHDC2 in which the YTH domain is responsible for recognition and binding to the m6A sites.^14^, ^15^

The previous studies have suggested that overexpression of the FTO has an oncogenic role in different cancer types^16^ such as acute myeloid leukemia,^17^ gastric cancer,^18^ cervical squamous cell carcinoma,^19^ and ESCC.^20^ It has been reported that down‐regulation of the FTO expression inhibits cell proliferation, migration and invasion abilities of ESCC cells.^20^ Recent studies have established, entacapone, a catechol‐o‐methyl transferase (COMT) inhibitor, can compete for the binding to the cofactor‐ and substrate binding sites on FTO and thereby can inhibit FTO activity.^21^ The entacapone, is the FDA approved drug which is currently in use for treatment of the Parkinson's disease in combination with levodopa.^22^ In this study, we aimed to investigate the potential effect of entacapone on m6a concentration and also its anti‐cancer effect on the cell viability and apoptosis of two different esophageal cancer cell lines, YM‐1 and KYSE‐30, in vitro.

## 2 METHODS

### 2.1 Reagent and cell line

In this study two ESCC cell lines were used. The YM‐1 cell line, which was previously established in the lab of Dr. Jahanbakhsh Asadi (Metabolic Disorders Research Center; Golestan University of Medical Sciences, Gorgan, Iran) from an ESCC female patient.^23^ The other cell line, KYSE‐30, was obtained from the cell bank of the Pasture Institute (Tehran, Iran). The cell culture reagents including the RPMI1640 culture medium, streptomycin/penicillin, EDTA Trypsin were purchased from Gibco, Life Technologies. Inc. The entacapone was provided as a gift from the Pharma Manufacturing at Fanda Pharma (Cat. No: ENT/516/06/20M;Rechem Lab). The m6A Elisa Kit (Cat.No:ZB‐15178c‐H9648; Zell Bio GmbH) and Nuclease P1 (Cat. No: Mo660s; BioLabs) and Alkaline phosphatase (P0114; sigmaaldrich) were purchased as indicated. annexin V/PI apoptosis detection kit was obtained from Mab Tag, GmbH.

### 2.2 Cell culture

In the present study, the two cell lines of human ESCC, YM‐1 and KYSE‐30, were cultured in RPMI‐1640 medium supplemented with 10% FBS and 1% streptomycin/penicillin. Cells were kept in a humidified incubator containing 5% CO_2_, at 37°C.

### 2.3 PBMC Isolation

The peripheral blood mononuclear cells isolation was performed using the ficoll reagent. Thus, 10 ml of peripheral blood containing anticoagulants was collected from a healthy person. The blood was diluted with equal volume of PBS and then was added to ficoll, in two separate layers. Then, to separate the PBMC containing layer, solution was centrifuged at 2500 rpm for 30 min. cells were rinsed by PBS. The peripheral blood mononuclear cells (PBMC) then were suspended in 1 ml of RPMI supplemented with 10% FBS and 1% streptomycin/penicillin and used for next experiments.

### 2.4 Viability assay

The toxicity of entacapone was evaluated in 48 h at different doses, in both esophageal squamous carcinoma cell lines, YM1 and KYSE 30, using MTT assay. Cells were seeded into 96‐well plates at a density of 1 × 10^4^ cells/well after 24 incubations, the culture medium was removed and then then cells were treated with different concentrations (25, 50, 75, 100, 125, and 150 μM) or IC50 (140 μM) of entacapone with a final volume of 200 μl. As vehicle control cells were treated with DMSO (0.3%) with same condition. After 48 h treatment, 10 λ of MTT assay (10 mg/mL) solution, (3‐(4,5‐dimethylthiazol‐2‐yl)‐2,5‐diphenyltetrazolium bromide), was added to each well and incubated at 37°C for 4 h. Then, the supernatant was aspirated off and 100 μl of DMSO was added to solve the precipitates. The absorbance of each well was measured at wavelength 570 nm using a micro‐plate reader. Also the viability was checked with dye exclusion assay using Trypan blue dye. Then the viable cell percentage was calculated as (trypan blue negative cell/total cell) × 100.

### 2.5 Apoptosis assay

To perform the apoptosis assay, 4 × 10^5^ cells were seeded in 6‐well plates. After 24 h cells were treated with 0.3% DMSO (control) and 140 μM entacapone for 48 h. Cells were stained by annexin‐V and propidium iodide (PI) at room temperature (RT) for 15 min, according to the manufacturer's protocol. The annexin V binds to phosphatidylserine (PS) exposed to the plasma membrane of cells undergoing apoptosis. This feature allows the living cell discriminated from early (stained only with annexin V) and late (stained with annexin V and PI) apoptotic cells. Briefly, 10^4^–10^6^ cells were re‐suspended in 90 μl of diluted binding buffer. After addition of 5 μl annexin V and 5 μl PI, the cell suspension was incubated for 20 min in the dark. Subsequently, 400 μl diluted binding buffer was added and then the supernatant was removed following centrifugation at 400*g* for 5 min. Finally the stained cells were re‐suspended in 200 μl diluted binding buffer and were analyzed by flow cytometry. The non‐stained cells also were used to distract the background in flow cytometer instrument. We counted the events for the living (annexin V−/PI−), necrotic (annexin V−/PI+), early apoptotic (annexin V+/PI−), and late apoptotic (annexin V+/PI+) cells using a BD Accuri™ C6 flow cytometer. The results were analyzed using the software supplied in the instrument.

### 2.6 RNA extraction

Total RNA was isolated using the RNX‐PLUSKit (SINACLON, Iran) according to the manufacturer's protocol. The number of 3 × 10^4^ cells of YM‐1 or KYSE‐30 cells were cultured in the flask. After 24 h incubation, cells were treated with DMSO (0.3% as control) or entacapone (IC50 = 140 μM) for 48 h. Then, cells were harvest with trypsin solution and washed twice with PBS. To extract the RNA, briefly, cells were disrupted and homogenized with RNX‐PLUS reagent at room temperature (RT) for 5 min. Then, chloroform was added to the samples and shacked vigorously for 5–10 s, then incubated for 15 min. Subsequently, using centrifugation (12 000 rpm at 4°C for 15 min), the upper aqueous phase containing the nucleic acids was isolated. Then, it was precipitated by adding equal volume of isopropanol followed with incubation for 15 min on ice and centrifugation. The RNA was washed twice by adding 1 ml 75% ethanol, and centrifugation at 7500 rpm at 4°C for 8 min. Finally, the purified RNA samples were dissolved in RNase‐free water and stored at −80°C.

### 2.7 Measurement of m6a by ELISA method

The level of m6A modification in RNA samples, was measured by m6 A Elisa Kit (Zellbio, Germany), following the manufacturer's protocol. Briefly, a total amount of 5 μg RNA for each group was used to determine the amount of m6A. To remove any RNA secondary structure, RNA samples were heated at 95°C for 5 min, and then was rapidly chilled on ice. The RNA was digested to nucleosides; by incubating the denatured RNA with 5 unit of nuclease P1 for 1 h at 37°C. Subsequently, 5 units of alkaline phosphatase plus sufficient of tris buffer to a final concentration of 100 mM Tris, pH 7.5 was added, and then was incubated for 1 h at 37°C. The supernatant was collected and was used for the ELISA experiment. The absorbance was measured at the wavelength 450 nm using a microplate reader. The standard curve was constructed to calculate the concentration of the samples.

### 2.8 Cell cycle assay

The cell cycle analysis was performed using flow cytometer with PI staining method. Labeling DNA with PI allows for fluorescence‐based analysis of cell cycle according the DNA content of each cell in different cell cycle phase (G1:2N, S:2N‐4N, G2/M = 4N). The number of 10^6^ cells were seeded in 25 cm^2^ flasks and allowed to attach for 24 h. Cells were then treated with DMSO (0.3%) and entacapone (140 μM) for 48 h. The cells were detached by trypsin and then fixed with 70% ethanol for 1 h. The cells were washed twice with PBS and then were incubated with stain solution containing 10 μg/ml propidium iodide and 40 μg/ml RNase in phosphate‐buffered saline and triton X‐100 (0.1%) for 30 min at 37°C. The percentages of cells in different phases of the cell cycle (G0/G1, S, and G2/M) can be quantified by BD Accuri™ C6 flow cytometry. The results were analyzed using the software supplied in the instrument.

### 2.9 Statistics

In this study the *p*‐value < .05 was considered as significant level. All the experiments were carried out in replicates (*N* = 3–5) for each group. For data visualization, the Graph Pad Prism v.5.04 was used and the data was reported as mean ± SE. for statistical analysis, SPSS v.19 was used. The normality of data was checked with the Shapirowik test and then in the case of normal distribution one‐way Anova test was used. In the case of non‐normal distribution the non‐parametric test was used for data analysis.

## 3 RESULTS

### 3.1 Entacapone has selective significant cytotoxicity in ESCC

**FIGURE 1:**
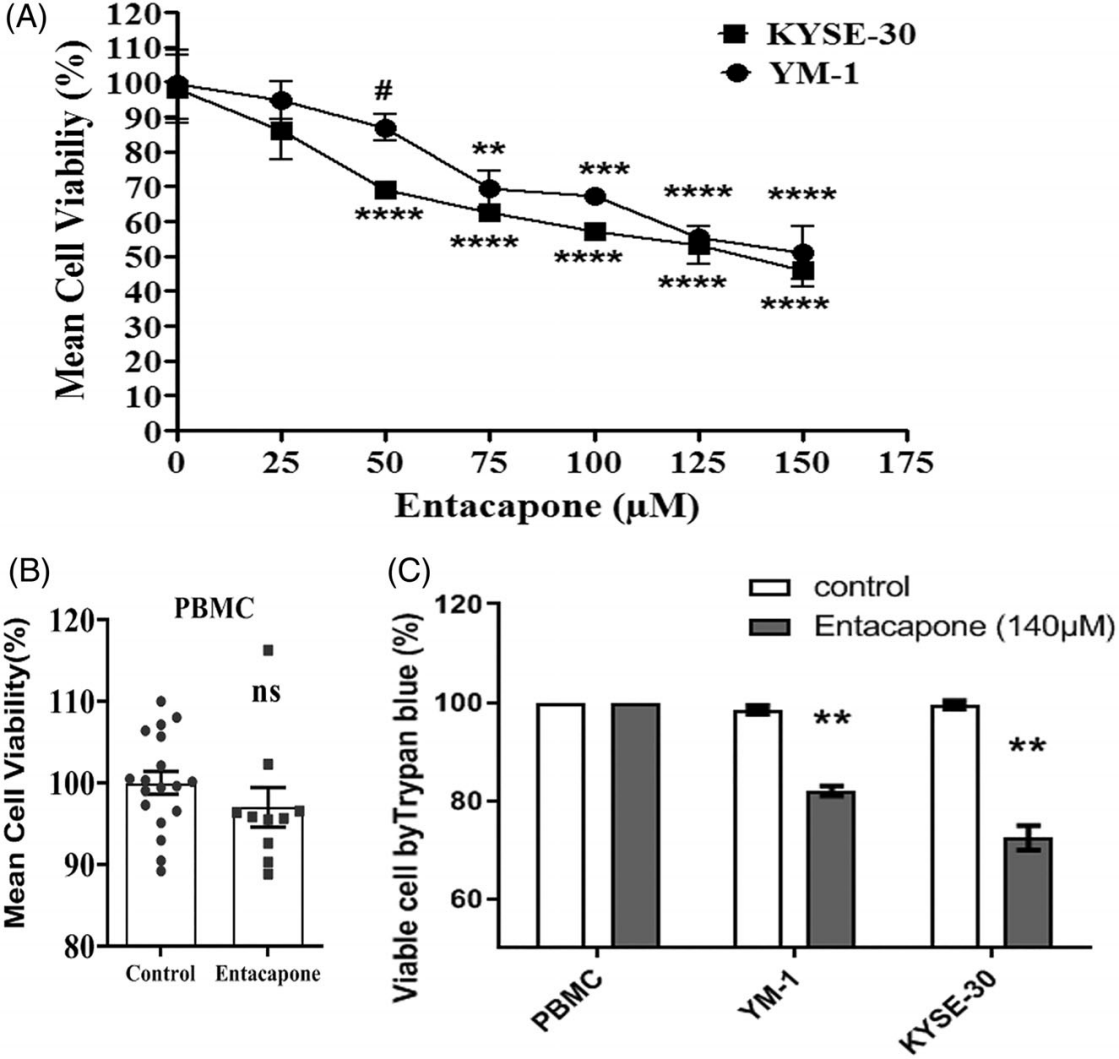
The cellular viability of two ESCC cancer cell lines (YM1 and KYSE‐30) and PBMC after entacapone treatment. Different concentrations of entacapone were used for treating cancer cells for 48 h, and the viability was measured using MTT. The cellular viability of YM1 and KYSE30 was compared to the control for each concentration (*N* = 3 in each concentration) (A). The viability of PBMC cells isolated from healthy as normal cells were also calculated (*N* = 18 replicates for control, *N* = 10 replicates for entacapone) (B). The viability of the cells was measured using trypan blue exclusion assay in control (DMSO 0.3%) and entacapone (140 μM) cells (*N* = 3 in each group) (C). * Represents the *p*‐value comparing to control which was calculated using the student *t*‐test. # shows *p*‐value < .01 YM‐1 vs KYSE‐30 at 50 μM entacapone

To evaluate the toxicity of entacapone in esophageal cancer cell lines (YM‐1 and KYSE30), cell viability was measured by MTT staining method for different concentrations. The percentage of cell viability after 48 h of treatment is shown in Figure 1A. Analysis of MTT data showed that different concentrations of entacapone inhibited cancer cell viability in both cell lines in comparison to the control (DMSO 0.3%). As shown in Figure 1A, the cytotoxicity of entacapone on YM‐1 and KYSE‐30 cells was started from 75 to 50 μM concentration, respectively. Our results showed a dose dependent cytotoxicity in both cell lines with declines cellular viability in higher concentrations. After 48 h of treatment, the IC_50_ value was calculated for YM‐1 (IC50 = 149 μM, *y* = −0.3558*x* + 103.99, *R* ^2^ = 0.9404) and KYSE‐30 (IC50 = 131.8 μM, *y* = −0.3347*x* + 93.592, *R* ^2^ = 0.9085) cell lines. For the next experiments, the mean IC_50_ value of two cell lines (140 μM) was used. When comparing the two cell lines together, the cytotoxic effect of entacapone was almost the same in both cell lines, however the KYSE‐30 showed more sensitivity in general with a significant difference with YM‐1 in concentration of 50 μM (Figure 1A). As a normal cell control, the PBMC, isolated from healthy individual was used. The viability was evaluated at a dose of 140 μM of entacapone after 48 h (mean IC_50_ for cancer cells). Our findings illustrated that the mean survival of PBMC cells treated with entacapone (Mean ± SE:100 ± 1.39) was not significantly different (*p*‐value: .693) from control group(Mean ± SE: 95.70 ± 2.56),indicating the selective toxicity of entacapone in cancer cells (Figure 1B). The dye exclusion assay using trypan blue also indicated selective toxicity of entacapone (140 μM) on YM‐1 (*p*‐value = .004) and KYSE‐30 cells (*p*‐value = .008) but no toxicity in PBMC was detected (Figure 1C).

### 3.2 Entacapone treatment induces apoptotic cell death in ESCC

**FIGURE 2:**
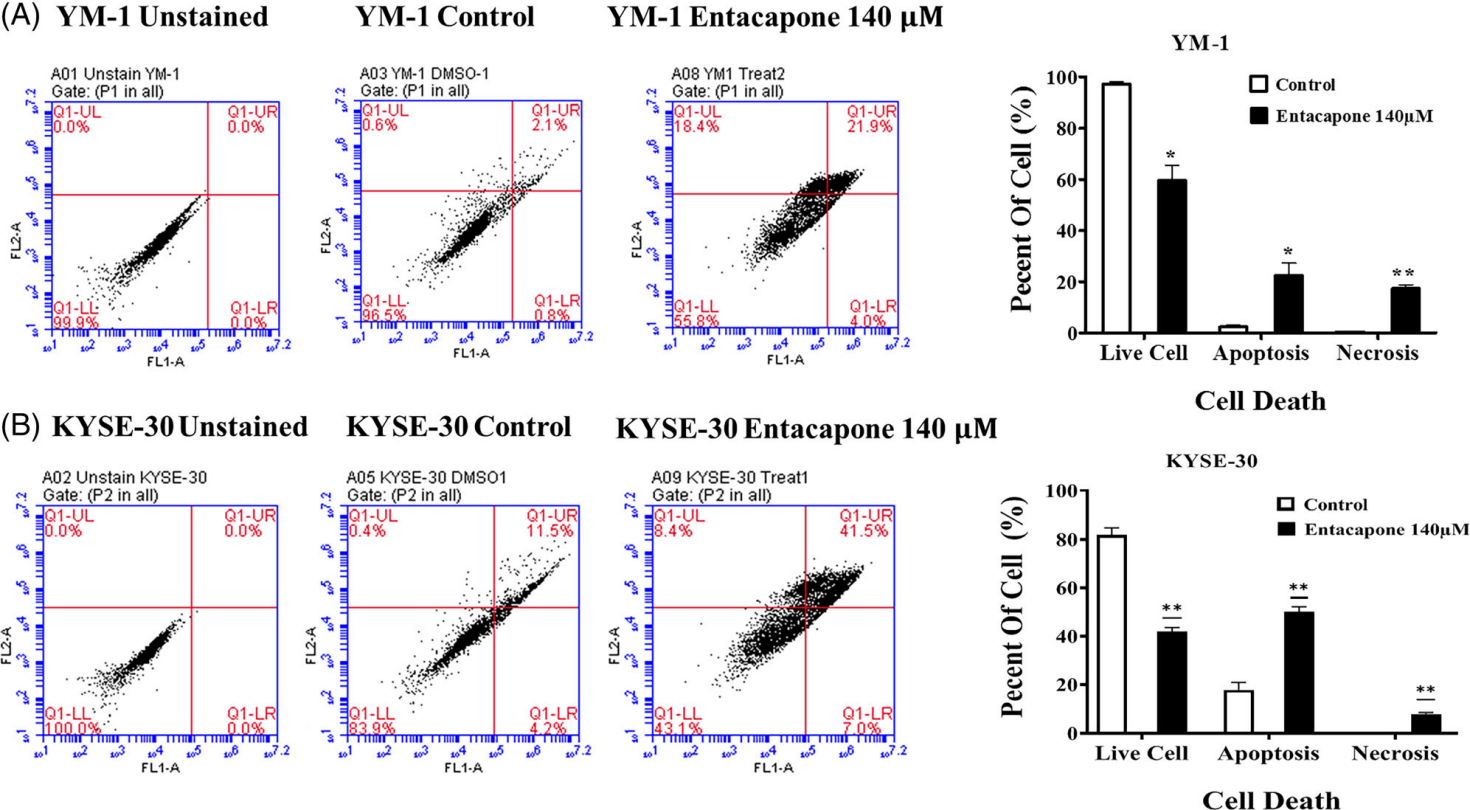
The apoptotic cell death in YM1 (A) and KYSE 30 (B) cell lines with entacapone treatment. The graph shows the dot plot of annexin (FL1‐A) and PI (FL2‐A) positive cells. The percentage of the live cells (double negative), apoptotic cells (early and late apoptotic), and necrotic cells (PI positive) were visualized in the bar chart for each cell line. Each bar represents mean of *N* = 3 replicates

The programmed cell death was measured using annexinV/PI staining protocol and flow cytometry technique. As shown in Figure 2A, B, the percentage of positive annexinV+/PI+ cells and positive PI cells treated with entacapone was significantly higher than the control in both cell lines. The entacapone was found to significantly increase the number of YM1 cells in the apoptotic phase (up to mean ± SE: 22.65 ± 3.25, *p*‐value = .02), as well as necrotic cells (up to mean ± SE = 17.55 ± 0.85, *p*‐value = .002) at mean IC50 concentration. Also, in the KYSE‐30 cell line, treatment with 140 μM entacapone resulted to increase in the apoptotic cells including both early and late apoptosis (up to mean% ± SE: 50.05 ± 1.55, *p*‐value = .009), as well as necrotic cells (up to mean% ± SE: 8.05 ± 0.35, *p*‐value = .002). There was no significantly higher number of early apoptotic phase at mean IC50 (140 μM) in KYSE‐30 versus control, while the number of cells in the early apoptotic phase increased significantly in YM‐1 (mean% ± SE: 3.8 ± 0.2 vs. 0.7 ± 0.10, *p*‐value = .0052. data not shown).

### 3.3 Measurement of m6A concentration in ESCC cell lines treated with entacapone

**FIGURE 3:**
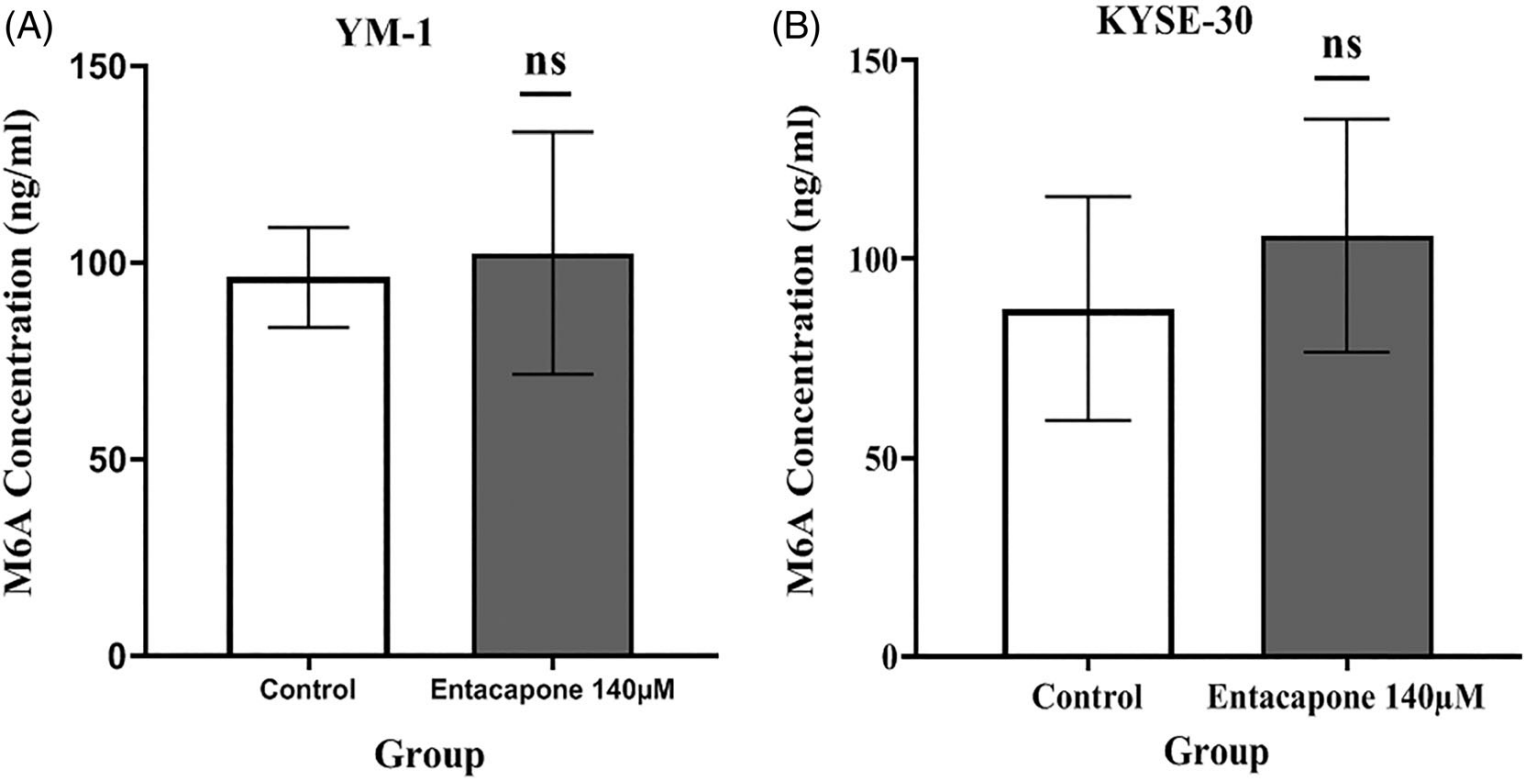
The concentration of m6A in total RNA of ESCC cells. Each bar represents the mean ± SE and ns: *p*‐value > .05. The control cell was treated with the DMSO 0.3% as vehicle control. Each bar represents mean of *N* = 3 replicates

The entacapone previously was introduced as inhibitor of FTO demethylase which removes the methyl group from m6A, then we hypothesized it may increase the m6A level as substrate of FTO. To test this hypothesis, the concentration of m6A in total RNA samples extracted from both cell lines was measured using ELISA in entacapone treated in comparison to the control cells. We found a small increase in m6A level in entacapone treated ESCC; however the change was not statistically significant (Figure 3).

### 3.4 Entacapone treatment changes the cell cycle progression in ESCC

**FIGURE 4:**
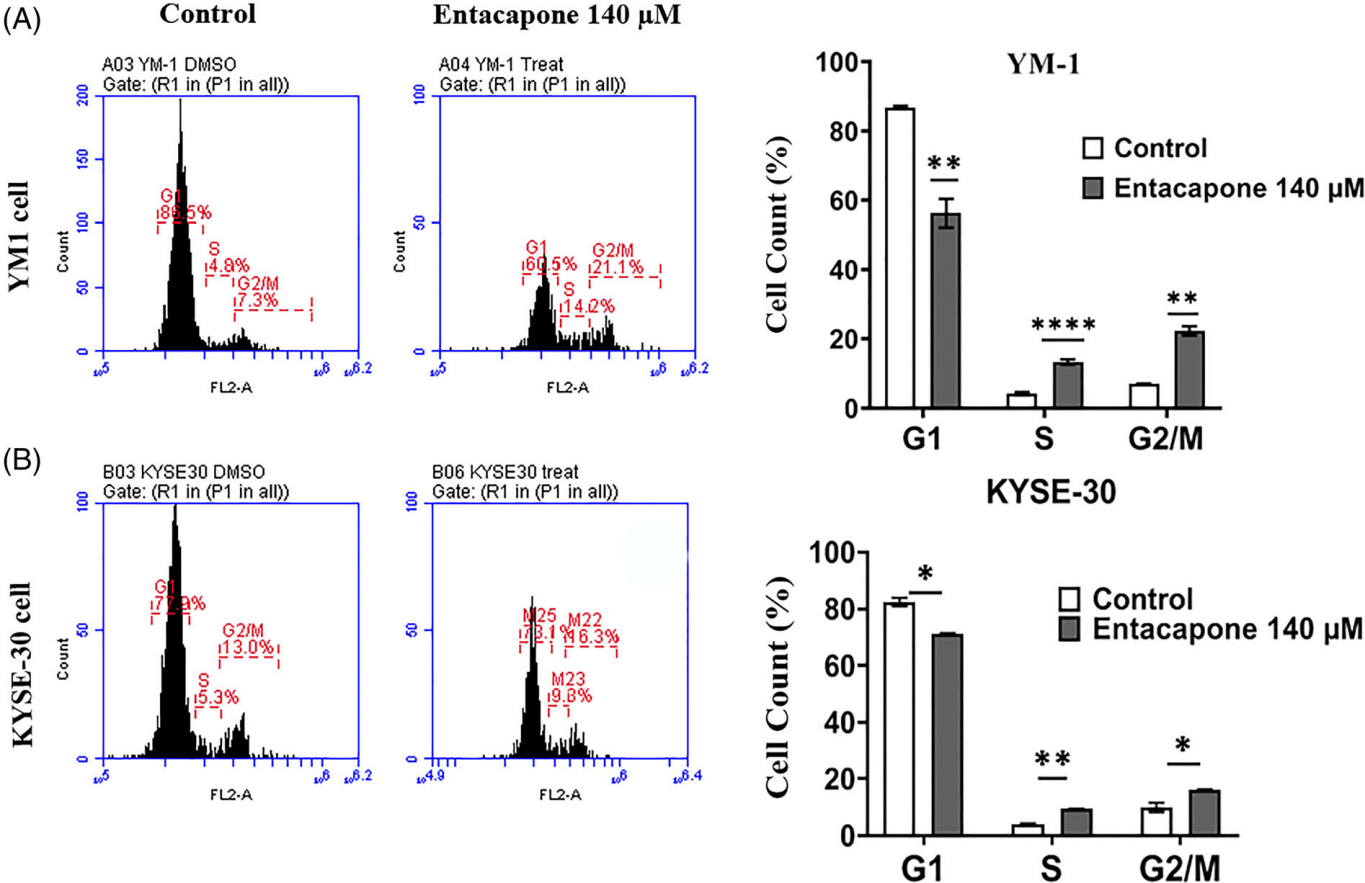
The cell cycle progression (G1, S, and G2/M) of YM‐1 and KYSE‐30 cells treated with entacapone. Each bar represents the mean ± SE and * shows the *p*‐value calculated using the student *t*‐test. Cells were treated with a mean IC50 concentration of entacapone (140 μM) for 48 h, and the control cell was treated with the DMSO 0.3% as vehicle control. Each bar represents mean of *N* = 3 replicates

For more investigation, the ESCC cells were stained for cell cycle using the PI and the cell cycle progression was measured by flowcytometery. Or findings showed that the entacapone treatment decreasescell in G1 phase (YM1:56.40 ± 2.36% vs. 86.95 ± 0.25%, *p*‐value = .005; KYSE30: 71.25 ± 0.20% vs. 82.35 ± 1.47%, *p*‐value = .016) while increasing the percentage of cells in S phase (YM1: 13.40 ± 0.46% vs. 4.35 ± 0.25%, *p*‐value = .000; KYSE30: 9.45 ± 0.086% vs. 3.95 ± 0.49%, *p*‐value = 0.007) and G2M phases of cell cycle in both cell lines (YM1:22.46 ± 0.77% vs. 7.05 ± 0.14%, *p*‐value = 0.002; KYSE30: 16.10 ± 0.23% vs. 9.95 ± 1.58%, *p*‐value = .058). This result shows a modulatory effect in cell cycle progression increasing cancer cells in S/G2M phase of cell cycle in both YM1 and KYSE 30 cells in vitro (Figure 4).

## 4 DISCUSSION

The esophagus squamous cell carcinoma is one of the most common types of esophageal malignancies with aggressive nature^24^ and poor survival rate.^20^ There are many factors playing role in ESCC pathogenesis, including genetic and epigenetic, as well as epitranscriptomic changes.^24^, ^25^ The role of epitranscriptome changes in cancer progression of different types has been investigated in recent years.^26^ The most common type of the epitranscriptome modification is N6‐methyladenosine (m6A) which can be modulated by methyltransferases METTL3/14, demethylases FTO and ALKBH5 and m6A‐binding proteins called YTHDF1‐3.^25^, ^27^

Increased expression of FTO as m6A demethylase has an oncogenic role in various types of cancers including ESCC. In recent years, the use of FO inhibitors as regulator of m6A, has been investigated as new potential therapeutic strategy for cancer. Available pharmaceutical compounds such as meclofenamic acid, nafamostet and eantacapone, in addition to siRNA and chemical compounds, have been introduced as FTO inhibitors.^21^, ^28^, ^29^, ^30^ Studies have shown that FTO enhances the proliferation and migration of esophageal cancer cells, and inhibiting FTO with siRNA potentially reduces the cell growth, proliferation, and migration of human esophageal cancer lines including KYSE150, Eca‐109 and TE‐1.^20^

In other hand a recent drug virtual screening introduced entacapone as a potential inhibitor of FTO which can directly bind to it and suppress its activity in vitro.^21^

Then here in current study we evaluated the potential anti‐cancer properties of the entacapone as FTO inhibitor in ESCC cells. We found a significant dose‐dependent toxicity of entacapone in both ESCC cells (YM‐1 and KYSE‐30) with the mean IC50 of 140 μM after 48 h of treatment without any significant toxicity in normal PBMC. Similar to our findings; Grimes et al. in 2018, showed the toxicity of entacapone alone (IC50 = 100 μM) or in combination with anthocyanin (IC50 = 50 μM), in colon (Caco‐2 and HT‐29) and Breast (MDA‐MB‐231) cancer cell lines after 72 h of treatment.^31^ In another study, Forester et al. in 2014, investigated the simultaneous effect of entacapone, tolcapone and epigallocatechin‐3‐gallate (EGCG) in human lung (H1299) and mouse lung (CL‐13) cancer lines. They reported the inhibitory concentration of IC50 = 76.8 μM and 50.7 μM after 72 h for the two cell lines respectively.^32^ The difference in IC50 value between their study and ours result could be related to the time of exposure and also the difference in cancer cell lines.

Our findings indicated that the 50% cellular death found in ESCC cells in response to the IC50 of the entacapone is through apoptosis with 22.65% ± 3.25 and 50.05% ± 1.55 of annexin/PI positive cells in YM‐1 and KYSE‐30 cell lines. Other studies also reported apoptotic cell death in acute myeloid leukemia cells treated with FTO shRNA^17^ and GC1 spermatogonia cell line treated with meclofenamic acid as FTO inhibitor.^33^

Regarding the inhibitory effect of entacapone on FTO we expected to observe significant increase in m6A as substrate of FTO, however unexpectedly our results showed a minor non‐significant increase in m6A of total RNA from entacapone treated ESCC (mean difference YM‐1 = +6 ng/ml and KYSE‐30 = +8 ng/mL). This could be explained by the complexity of m6A regulation through different regulators including demethylase, methyl transferases and also m6A‐binding proteins.

Although overexpression of FTO demethylasehas been reported in esophagus tumor in associated with lower survival rate and poorer prognosis^20^; but overexpression of METTL3 methyl transferases,^34^ and ALKBH5 as another m6A demethylase^35^ is also reported to be associated with poor prognosis in esophageal tumors. So the other m6A modulators may potentially affect the m6A level compensating the effect of entacapone in FTO demethylase inhibition. Also the cytotoxic effect of entacapone can be related to other mechanisms independent to FTO or m6A modulation.

Moreover we found a significant change in the cell cycle progression of entacapone treated ESCC cells by decreased G1 and S, G2/M increase. Nagaki et al reported a cell cycle delay with G0/G1 arrest in ALKBH5 siRNA treated ESCC cell lines through up‐regulation of the CDKN1A (p21) consequent to increased m6A and stability of its mRNA.^35^ In another study it was reported that entacapone or tolcapone in combination with EGCG can reduce expression of cyclin D1 and consequently, resulting to G1 arrest induction in H1299 cells, whereas in CL‐13 lung cancer cells they found significant G2/M arrest, which was in parallel to our findings.^32^ Similar studies support the role of the FTO in cell cycle progression through regulation of cyclin D1 m6A modification. It has been reported that FTO inhibition can prolong G1 phase by cyclin D1 suppression.^36^ Altogether although there are various effects of FTO siRNA, or chemical compounds in cell cycle progression in different cell lines, but all of these share common outcome in general in cell cycle inhibition.

## 5 CONCLUSION

Our findings indicated selective anti‐cancer effect of entacapone in esophageal cancer cells in vitro through apoptosis induction and cell cycle regulation. These results suggest potential application of entacapone in ESCC treatment however further investigations to discover the underlying mechanisms are needed.

## AUTHOR CONTRIBUTIONS

**Fahimeh Ramedani:** Investigation (equal); writing – original draft (equal); writing – original draft (equal). **seyyed mehdi Jafari:** Investigation (equal); supervision (equal). **Marie Saghaeian Jazi:** Investigation (equal); methodology (equal); writing – review and editing (equal). **Zeinab Mohammadi:** Investigation (equal); writing – review and editing (equal). **Jahanbakhsh Asadi:** Project administration (equal); supervision (equal); validation (equal).

## FUNDING INFORMATION

This project was extracted from a master student thesis which was financially supported by Golestan University of Medical Sciences (grant number: 111290).

## CONFLICT OF INTEREST

The authors declare that there are no conflicts of interests.

## ETHICS STATEMENT

This project was approved by ethical committee of Golestan University of Medical Sciences (Approval code: IR.GOUMS.REC.1398.357).

## Data Availability

The data that support the findings of this study are available from the corresponding author upon reasonable request.
